# Relationship between MRI-based mechanical activation delays and direct intraoperative measurements of electrical delay in patients undergoing cardiac resynchronization

**DOI:** 10.1186/1532-429X-15-S1-P63

**Published:** 2013-01-30

**Authors:** Jonathan Suever, Gregory R Hartlage, Shahriar Iravanian, Michael Lloyd, John N Oshinski

**Affiliations:** 1 Wallace H. Coulter Department of Biomedical Engineering, Georgia Institute of Technology / Emory University, Atlanta, GA, USA; 2 Division of Cardiac Electrophysiology, Department of Medicine, Emory University School of Medicine, Atlanta, GA, USA; 3 Department of Radiology and Imaging Sciences, Emory University School of Medicine, Atlanta, GA, USA

## Background

Magnetic Resonance Imaging (MRI) can be used to create maps of left ventricular (LV) mechanical activation delay by analyzing time-dependent radial motion of the myocardium. Local electrograms (EGM) can be obtained during the CRT procedure to measure local electrical activation delay in the LV myocardium. Preoperative MRI assessment of regional dyssynchrony would be useful to guide CRT lead placement. However, the relationship between electrical and mechanical dyssynchrony has not been studied in humans. We hypothesized that intraoperative electrical delay times will correlate with MR-based mechanical delay times.

## Methods

This study compared 24 intraoperative LV lead sites in 7 patients undergoing CRT that met standard inclusion criteria (QRS Duration>120ms, NYHA HF Class III-IV, EF<35%).

**Figure 1:**
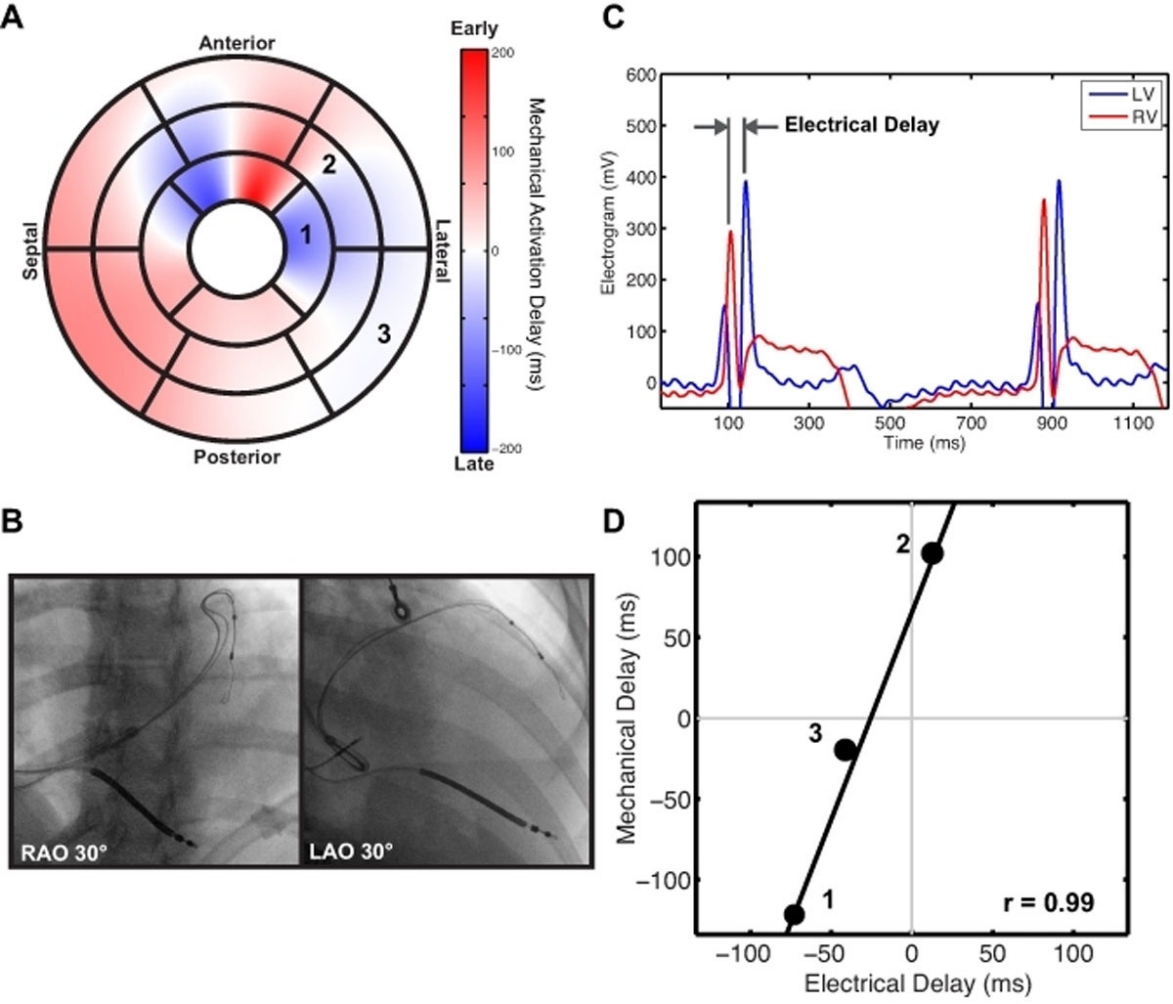
Mechanical delay times from MRI data were mapped to the AHA 17-segment model (A). A bipolar lead was advanced to several locations throughout the coronary veins (B) and electrograms were obtained at each location(C). The lead locations were mapped to the AHA 17-segment model (numbers) and mechanical and electrical delay times were compared (D).

MR cine short-axis SSFP images were acquired with 60 frames per cardiac cycle. Radial displacements of the endocardium were used to generate maps of regional mechanical delay times (Figure 1A) using an existing technique (Suever, et al. SCMR 2012).

During the CRT implantation procedure, the right ventricular (RV) pacing lead was positioned in the RV apex. The LV pacing lead electrodes were positioned in several sites within the coronary venous system. At each location, bi-plane X-rays were acquired and local EGMs were obtained for both the RV and LV leads (Figure 1B). The electrical activation delay time was defined as the peak-to-peak difference between the LV electrode EGM and the fixed reference RV EGM (Figure 1C).

LV lead recording positions were mapped to the AHA bullseye by an experienced observer. Mechanical activation delays in the region surrounding each lead location were compared to the corresponding mechanical and electrical delay times (Figure 1D).

## Results

EGMs were successfully acquired at 24 locations throughout the coronary veins in 7 patients. Intraoperative electrical delay times were -40.0 ± 61.3 ms and MR-derived mechanical delay times were 25.3 ± 71.1 ms. In each patient, mechanical and electrical delay times were strongly correlated with a mean r-value of 0.94 (range: 0.83-1.00). These data indicates that electrical delay is strongly coupled to mechanical delay as measured by MR.

## Conclusions

Mechanical activation delay times obtained using short-axis MRI data correlated on a patient-by-patient basis with invasive measurements of electrical activation delays in the LV. Not only does this provide support for using short-axis MRI for the detection of mechanical dyssynchrony, but also provides the opportunity to study electromechanical coupling in CRT patients.

## Funding

Funding for this research was provided by AHA Grant-in-Aid, the National Science Foundation Graduate Research Fellowship Program, and by the National Center for Advancing Translational Sciences of the National Institutes of Health Award Number UL1TR000454.

